# Working together in health research: a mixed-methods patient engagement evaluation

**DOI:** 10.1186/s40900-023-00475-w

**Published:** 2023-08-01

**Authors:** Stella Babatunde, Sadia Ahmed, Maria Jose Santana, Ingrid Nielssen, Sandra Zelinsky, Anshula Ambasta

**Affiliations:** 1 Department of Pediatrics, Cumming School of Medicine, University of Calgary, Calgary, AB Canada; 2 Department of Community Health Sciences, Cumming School of Medicine, University of Calgary, Calgary, AB Canada; 3 Alberta Strategy for Patient-Oriented Research (AbSPORU) Patient Engagement Team, Calgary, Canada; 4 Department of Anesthesiology, Pharmacology and Therapeutics, Faculty of Medicine, University of British Columbia, Vancouver, BC Canada

**Keywords:** Patient engagement, Patient research partners, Patient-oriented research, Patient advisory council

## Abstract

**Background:**

In patient-oriented research (POR), patients contribute their valuable knowledge and lived-experiences to work together as active research partners at all stages of the health research cycle. However, research looking to understand how patient research partners (PRPs) and researchers work together in meaningful and collaborative ways remains limited. This study aims to evaluate patient engagement with the RePORT Patient Advisory Council (PAC) and to identify barriers and facilitators to meaningful patient engagement encountered within research partnerships involving patient research partners and researchers.

**Methods:**

The RePORT PAC members included nine PRPs and nine researchers (clinician-researchers, research staff, patient engagement experts) from both Alberta and British Columbia. All members were contacted and invited to complete an anonymous online survey (Public and Patient Engagement Evaluation (PPEET) tool) at two different project times points. The PAC was invited for a semi-structured interview to gain in-depth understanding of their experiences working together. Interviews were audio-recorded, transcribed, and the data was thematically analyzed with the support of a qualitative analysis software, NVivo.

**Results:**

A total of nine PRPs (100%) and three researchers (33%) participated in the baseline survey in February 2022 while six PRPs (67%) responded and three researchers (33%) completed the follow up survey in May 2022. For the semi-structured interviews, nine PRPs (100%) and six researchers (67%) participated. According to the survey results, PAC members agreed that the supports (e. g. training, compensation) needed to contribute to the project were available throughout the project. The survey responses also showed that most members of the PAC felt their opinions and views were heard. Responses to the survey regarding diversity within the PAC were mixed. There were many suggestions for improving diversity and collaboration provided by PAC members during the semi-structured interviews. PAC members mentioned that PAC PRPs informed the co-development of research materials such as recruitment posters and interview guides for the RePORT study.

**Conclusions:**

Through fostering a collaborative environment, we can engage a diverse group of people to work together meaningfully in health research. We have identified what works well, and areas for improvement within our research partnership involving PRPs and researchers as well as recommendations for POR projects more broadly, going forward.

**Supplementary Information:**

The online version contains supplementary material available at 10.1186/s40900-023-00475-w.

## Abstract (plain language summary)

Patient research partners contribute their valuable knowledge, lived experiences, and skills in health research projects. However, research looking to understand how patient partners and researchers can work together in a meaningful and collaborative way remains limited. We aim to evaluate patient engagement with the RePORT Patient Advisory Council (PAC) and to identify barriers and facilitators to meaningful patient engagement encountered within research partnerships involving patient research partners and researchers. We used a mixed methods design as it provided the opportunity to gather more insights from the RePORT PAC members’ engagement experiences throughout the study and provide a deeper understanding on the barriers and facilitators to working together. We involved diverse patient research partners, clinicians/researchers, patient engagement organization team members and other stakeholders from the RePORT PAC. All PAC members were invited to complete an anonymous online survey as well as a semi-structured interview. Patient research partners appreciated the supports (e.g. training, compensation) provided throughout the project. Most PAC members felt that their views and opinions were heard, which further facilitated a collaborative team environment. There were many suggestions for improving diversity and collaboration provided by PAC members during the semi-structured interviews. Through fostering a collaborative environment, we can engage a diverse group of people to work together meaningfully in health research. We have identified what works well, and areas for improvement in our research partnership and recommendations for patient-oriented research projects more broadly, going forward.

**Supplementary Information:**

The online version contains supplementary material available at 10.1186/s40900-023-00475-w.

## Background

The process of including patients as partners who bring experience-based knowledge to health research teams has become increasingly recognized as valuable to conducting research that will meaningfully impact patients, families, and communities [1–3]. Recently, patients are involved as active and equal research partners in various research projects and activities, collaborating and contributing their knowledge, insights, and understandings at every stage of the research process [4]. Patient research partners (PRPs) can help to identify priorities, challenge assumptions, share critical insights and knowledge of living with a specific health condition and navigating the healthcare system. This can ultimately lead to research with greater impact, validity, quality of results, and relevancy for all end-users [5, 6].

The Strategy for Patient-Oriented Research (SPOR) initiative was established by the Canadian Institutes of Health Research (CIHR) to develop key concepts, principles, and areas for patient engagement [6]. SPOR defines patient engagement as the meaningful and active collaboration of patients in all areas of the research project including governance, priority setting and development of research question [6]. The role of patients in research can differ depending on the project; patients can be advisors, collaborators, co-researchers, contributors or supporters [6, 7]. In addition to lived experience expertise, PRPs bring other skills, and knowledge that could be valuable to the research.

In patient-oriented research (POR), patients and caregivers are involved as partners throughout the research process with the focus on the priorities important to the patients to ultimately improve health care system and delivery [7, 8]. In recent years, there has been an increased focus on research exploring the meaningful and collaborative interactions between PRPs and researchers. A scoping review of 55 research articles in Canada, the United States and the United Kingdom showed successful patient engagement approaches and opportunities [9]. However, the authors noted that the uptake of patient engagement in health research is slow, and outcomes are unclear [9]. The authors also reported the need for clarification of patient engagement terminology and support for development of patient engagement evaluation frameworks and tools [9]. Few studies have formally evaluated the patient engagement processes and the impact on PRPs and project outcomes. In a study involving patients as partners in primary care research, PRPs felt they had a significant impact on the study process and outcomes [2]. Another study showed that patient engagement was valued and involving patients increased the validity of the findings as well as promoted learning and transparency within the research [10]. In a systematic review conducted by Brett et al. [11], the authors found that patient and public involvement has positive impacts on service users, researchers, and patient groups despite challenges. There is a wide range of patient engagement evaluation tools and frameworks available [12–14]. In the review by Dukhanin et al. [12], patient engagement tools were identified; however not all of them were used for health research, and not all were validated measures [12].

SPOR SUPPORT Units across Canada are funded by CIHR to provide services to health research teams on how to conduct POR and advance the engagement of patients as partners in research [15]. The Re-Purposing the Ordering of Routine laboratory Tests in hospitalized medical patients (RePORT) study project is one of the POR projects supported by the Alberta SPOR SUPPORT Unit (AbSPORU) Patient Engagement Team. RePORT is a collaborative project deployed in Alberta and British Columbia and aims at reducing the overuse of daily repetitive laboratory testing in hospitalized medical patients in the provinces. The Patient Advisory Council (PAC) was established to inform and contribute to all phases of this POR project. The RePORT PAC includes PRPs, clinician-researchers, research staff, and AbSPORU Patient Engagement team members.

The overall aim of this study was to evaluate the patient engagement process and impact within the RePORT PAC. Specifically, the objectives of the evaluation study were (1) to evaluate the approaches to patient engagement through the PAC in the RePORT study (2) to identify barriers and facilitators to engagement within the RePORT PAC and in future POR.

## Methods

### The RePORT PAC

The RePORT PAC was formed in May 2021. The RePORT PAC had an open membership policy and maintained a relatively stable membership since October 2021 after seeing some members leave due to competing priorities (e.g., health states, job commitments) early on. The RePORT PAC members at the time of the evaluation included nine PRPs, and nine researchers (three clinician-researchers, four research staff and two AbSPORU Patient Engagement team members) representing multiple health, health research, and educational backgrounds and perspectives, socio-economic, cultural, and geographic backgrounds from both Alberta and British Columbia. The AbSPORU Patient Engagement team members included a Patient Research Partner Lead who was also a co-investigator on the RePORT Grant, a Patient Engagement Coordinator, a Research and Evaluation Coordinator, and a Research Assistant. The RePORT PAC included the active participation of both the Patient Research Partner Lead and the Patient Engagement Coordinator.

There were four men and sixteen women in the PAC. A wide range of ages was represented among the PAC members. The PRPs within the PAC consisted of individuals of diverse gender identities, including both men and women. Some of the PRPs had previously served on other advisory councils and research teams. The patient engagement process was guided by the CIHR SPOR patient engagement framework which is based on four key principles: inclusiveness, mutual respect, support, and co-build [16]. The PAC met monthly to advise and inform on the RePORT study priorities and processes including the study recruitment strategy, study documents (e.g., providing input on the recruitment flyer, interview guide), and providing additional insights to the data analysis and findings. In addition, PRPs were offered opportunity to collaborate on additional project research activities including data collection, analysis, and knowledge translation. As per CIHR SPOR, training and support from the AbSPORU Patient Engagement and research team members were offered to make this engagement meaningful and equitable. Smaller working groups were formed, and meetings were scheduled in between the regular scheduled monthly PAC meetings. To supplement emails, the team had a shared Google drive to house meeting agendas, notes, and related documents and to facilitate communication outside of meetings. Monthly meetings were held virtually using Zoom, an online video conference platform. At each PAC meeting, there was at least 50% of members present. PRP attendance at each of the research skill-building workshops was more than 50% of the PAC membership. The PAC actively engaged in numerous activities throughout the RePORT project, encompassing the development of essential documents such as the Terms of Reference Document, ethics application, study protocol design, informed consent form, recruitment poster for patient participants, and interview guide. Additionally, the PAC members, particularly the PRPs, played crucial roles in facilitating interviews, developing a codebook for content analysis, rapidly analyzing transcripts, and creating a patient-engagement strategy focused on the appropriate utilization of laboratory testing for hospital patients.

The AbSPORU Patient Engagement Research and Evaluation Coordinator and Research Assistant conducted the evaluation study and therefore did not participate in the evaluation.

### Study design

A sequential mixed-methods approach using both qualitative and quantitative methods was used to evaluate the patient engagement within the PAC. The study followed a sequential explanatory mixed method design which consists of a quantitative data collection phase followed by a qualitative data collection phase [17]. We first collected quantitative survey data from RePORT PAC members to allow them to share their views and help us understand their assessment of key features of the patient engagement approaches, including perceptions on communication, supports and training for equitable and meaningful engagement. Additionally, we sought to understand the overall influence and impact of the patient engagement initiative on the PRPs involved in the research project. Subsequently, we conducted interviews to gain in-depth understanding of the barriers and facilitators to working together in the RePORT project. A mixed method approach provided the opportunity to gather more insights from the RePORT PAC members’ engagement experiences throughout the study and provide a deeper understanding on the barriers and facilitators to working together.

### Participants and survey tool

All members of the PAC were contacted via email and invited to complete an anonymous online survey using the validated Public and Patient Engagement Evaluation Tool (PPEET) [18]. Developed in a collaborative process by researchers and public and patient engagement practitioners led by McMaster University, the PPEET consists of a series of questionnaires to evaluate patient engagement from the perspectives of patient partners/advisors, staff, and organizational leaders [18]. A systematic assessment was conducted to evaluate the appropriateness and feasibility of PPEET (Patient and Public Engagement Evaluation Tool) for implementation in various health system settings. The findings indicated that PPEET was valuable, user-friendly, and reasonably comprehensive in its application [19]. While the tool was designed primarily to evaluate public and patient engagement within health system organization change and quality improvement projects, this tool has also been used in the context of health research. In addition to its appropriateness, the PPEET was selected for its convenience in terms of usability and administration through an electronic tool.

The survey was administered to all PAC members twice during the study (early February 2022, and in May 2022) with reminders sent out periodically. We summarized categorical responses in the survey into frequencies and graphs. Likert scales were used for categorical responses, with 1 representing strongly disagree and 5 representing strongly agree. Survey results were collected and summarized using Qualtrics, an online survey tool.

### Qualitative interview

After delivering the second survey, semi-structured interviews were conducted by a qualitative researcher (SB), using an interview guide that was co-developed in collaboration with PRPs from Albertans4HealthResearch [20], and researchers from the RePORT PAC. The interview guide was iteratively developed and refined based on the survey responses received from the first survey. The interviews ranged from approximately 30 min to one hour. The aim of the interviews was to gain an in-depth understanding of the barriers and facilitators to meaningful patient engagement approaches identified in the survey. A semi-structured interview consists of open-ended questions to gather in-depth narratives of individual thoughts, feelings, and experiences of working together on the team [21]. The interviewer is a student researcher from AbSPORU Patient Engagement completing a master’s in public health with extensive training in qualitative research. The interviewer had no prior experience with the PAC or RePORT project and was not an active member of the PAC. Interviews were audio-recorded and transcribed verbatim by the qualitative researcher who conducted the interviews. Transcripts were not returned to participants for review.

### Data analysis

Thematic analysis is a standard qualitative analysis method that consists of gathering, identifying and analyzing repeating patterns within a data set [22]. We followed the guidelines for thematic analysis outlined by Braun and Clarke (2008) [22]. Transcribing and reading the data were the first steps in understanding the data. Interview transcripts were coded first by the interviewer (SB), and then discussed with another team member (SA), trained in qualitative research analysis in multiple peer debriefing sessions. NVivo 12 was used to organize transcripts and codes, as well as to develop a code book [23]. We identified the initial codes and then grouped them into themes. These themes were further refined and defined. Finally, we identified the main overarching themes and organized the sub-themes within each category. In addition to specific answers to the interview guide questions, we also looked for interesting comments, suggestions, or responses. Data saturation, as predetermined by the authors, was reached when 100% of PRPs and 67% of researchers participated in the interviews; no additional data is being reviewed as part of the study.

The data including the interview file, transcripts and notes were stored in an encrypted, password-protected, secure network.

### Ethics

The University of Calgary Conjoint Health Research Ethics Board (CHREB) approved the evaluation study (REB20-1822).

## Results

**Fig. 1:**
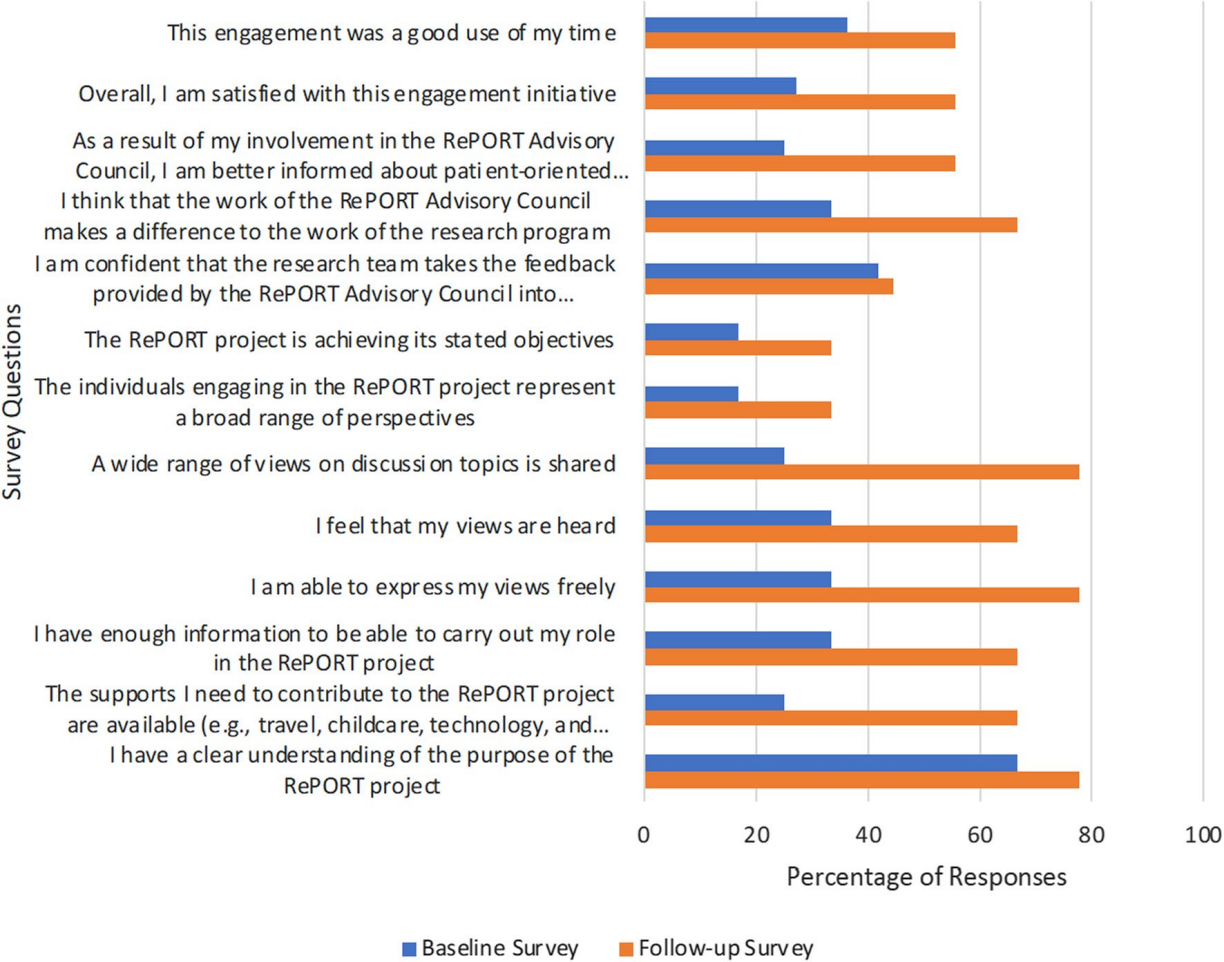
Comparison of responses for “strongly agree” in baseline vs follow-up surveys

A total of nine PRPs (100% response rate) and 3/9 researchers (33% response rate) participated in the baseline survey. This survey was conducted at the 3-month point of establishment of stable membership within the PAC. 6/9 PRPs (67% response rate) responded and completed the follow-up survey. There were 3/9 researchers (33% response rate) that responded and completed the follow-up survey. The follow-up survey was conducted a year after the PAC was initiated. The results from the initial and follow up surveys are included in Tables 1 and 2. The survey results indicated improved responses, with all questions showing an increased number of respondents strongly agreeing to the survey questions between the baseline and follow-up surveys (Fig. 1).

**Table 1:** Results from the Patient Engagement Evaluation Baseline Survey (n = 12)

	Strongly agree n (%)	Somewhat agree n (%)	Neither agree nor disagree n (%)	Somewhat disagree n (%)	Strongly disagree n (%)
I have a clear understanding of the purpose of the RePORT project	8 (66.7)	4 (33.3)	–	–	–
The supports I need to contribute to the RePORT project are available (e.g., travel, childcare, technology, and internet support)	3 (25.0)	9 (75.0)	–	–	–
I have enough information to be able to carry out my role in the RePORT project	4 (33.3)	6 (50.0)	2(16.7)	–	–
I am able to express my views freely	4 (33.3)	8 (66.7)	–	–	–
I feel that my views are heard	4 (33.3)	8 (66.7)	–	–	–
A wide range of views on discussion topics is shared	3 (25.0)	7 (58.3)	2 (16.7)	–	–
The individuals engaging in the RePORT project represent a broad range of perspectives	2 (16.7)	6 (50.0)	3 (25.0)	1 (8.3)	–
The RePORT project is achieving its stated objectives	2 (16.7)	8 (66.7)	2 (16.7)	–	–
I am confident that the research team takes the feedback provided by the RePORT Advisory Council into consideration	5 (41.7)	7 (58.3)		–	–
I think that the work of the RePORT Advisory Council makes a difference to the work of the research program	4 (33.3)	5 (41.7)	3 (25.0)	–	–
As a result of my involvement in the RePORT Advisory Council, I am better informed about patient-oriented research	3 (25.0)	6 (50.0)	2 (16.7)	–	–
Overall, I am satisfied with this engagement initiative	3 (27.3)	8 (72.8)	–	–	–
This engagement was a good use of my time	4 (36.4)	6 (54.5)	1 (9.1)	–	–

**Table 2:** Results from Patient Engagement Evaluation Follow-up Survey (n = 9)

	Strongly agree n (%)	Somewhat agree n (%)	Neither agree nor disagree n (%)	Somewhat disagree n (%)	Strongly Disagree n (%)
I have a clear understanding of the purpose of the RePORT project	7 (77.8)	2 (22.2)	–	–	–
The supports I need to contribute to the RePORT project are available (e.g., travel, childcare, technology, and internet support)	6 (66.7)	3 (33.3)	–	–	–
I have enough information to be able to carry out my role in the RePORT project	6 (66.7)	3 (33.3)	–	–	–
I am able to express my views freely	7 (77.8)	2 (22.2)	–	–	–
I feel that my views are heard	6 (66.7)	2 (22.2)	1 (11.1)	–	–
A wide range of views on discussion topics is shared	7 (77.8)	1 (11.1)	1 (11.1)	–	–
The individuals engaging in the RePORT project represent a broad range of perspectives	3 (33.3)	2 (22.2)	2 (22.2)	–	2 (22.2)
The RePORT project is achieving its stated objectives	3 (33.3)	6 (66.7)	–	–	–
I am confident that the research team takes the feedback provided by the RePORT Advisory Council into consideration	4 (44.4)	3 (33.3)	2 (22.2)	–	–
I think that the work of the RePORT Advisory Council makes a difference to the work of the research program	6 (66.7)	2 (22.2)	1 (11.1)	–	–
As a result of my involvement in the RePORT Advisory Council, I am better informed about patient-oriented research	5 (55.6)	4 (44.4)	–	–	–
Overall, I am satisfied with this engagement initiative	5 (55.6)	4 (44.4)	–	–	–
This engagement was a good use of my time	5 (55.6)	4 (44.4)	–	–	–

Nine PRPs (100%) and 6/9 researchers (67%) participated in the semi structured interviews. There were two men and thirteen women that participated in the interviews. Data analysis of the semi-structured interviews and surveys resulted in the identification of five main themes. The themes that were identified in this study were: (1) communication with team members about project (2) team dynamics impacting engagement (3) supports for PRPs to contribute (4) impact of PRPs on the project processes (5) improving the diversity of the council.

PAC members suggested ways to improve the relationship between PRPs and the rest of the PAC team. The PRPs are the patient research partners in the PAC while the researchers are the clinician-researchers, research staff and the AbSPORU Patient Engagement team members. The barriers and facilitators to engagement in the PAC as well as recommendations to other research teams working with PRPs are indicated in Table 3. Table 3 was created based on the interview responses, encompassing perspectives of both the RePORT PAC PRPs and researchers. Additional file 1: Table 4 provides an in-depth look at the themes identified in the interviews as well as supporting quotes [See Additional file 1]. The themes from both the interviews and survey results are described below.

**Table 3:** PAC members perspectives on barriers and facilitators to patient engagement in the RePORT project

Barriers encountered by the PAC	Facilitators identified by the PAC
The virtual environment made it difficult to communicate and engage	There were meaningful engagement opportunities for PAC members
Recruitment of diverse PAC members	The variety of perspectives on the PAC enhanced the research project. Monthly meetings were facilitated by a PRP chair to make sure that all voices were heard during these meetings
The difficulty of managing and scheduling a large group especially in different provinces and time zones	Flexibility of the project in terms of time commitment and what tasks to be involved in
Technical issues related to information sharing	Virtual meetings provide an opportunity for members from different locations (provinces) to meet
Clarity of roles within the project especially at the beginning	Communication within the team was good and created a collaborative environment. Challenges were addressed together as a team as they arose
Expectations of involvement in project were not always met for PRP	Capacity building opportunities for PRPs (workshops, training, mentorship) Contributions from PRPs were valued

Recommendations to other research teams working with PRPs	
Offer capacity development opportunities for PRPs	
Offer compensation to PRPs	
Provide flexibility in the involvement of PRPs	
Include PRPs from the beginning of project to foster patient engagement	
Use established guidelines and procedures for engaging PRPs	
Include PRPs from diverse backgrounds including ethnicity, gender, language, abilities etc	
Involve PRPs as peer mentors and for recruitment initiative	
Encourage open communication	

*PAC* advisory council; *PRP* patient research partner

### Communication with team members about project

Among PAC members, two sub-themes were identified: role clarity and information sharing. The baseline survey showed that 4/12 (33%) of respondents strongly agreed that they had enough information to understand their role on the team, while 6/9 (67%) strongly agreed in the follow-up survey.

In the interviews, a few PAC members expressed confusion about their roles as well as the roles of other team members when they initially joined the PAC *“I will say at the beginning of this project starting I found it to be really disorganized in the beginning because it wasn't clear who was responsible for what Uhm, and so I think it took a while for the team to kind of figure out…” (PRP8).*

Most PAC members who responded to the survey felt that the project details and instructions were clearly communicated throughout the project. One PAC member shared that communication was clear within the team *“I think the instructions on what they require and what they hope people to do whether you're an advisor, patient, advisor, or research or whatever, I think it's quite clear” (Researcher05).*

Amongst the facilitators of engagement in the PAC, one researcher shared that there was open communication amongst the PAC members. *“there’re nine patient research partners in total like I think it's like at least 6 or 7 of them have been involved in everything like engaged with everything so the PAC, the research, all different stages of the research. And so, by having them so …heavily involved … I get the sense that most of them are very comfortable talking and sharing their thoughts and opinions, suggestions, their insight, *etc*. So, through the different phases, they were always like helping us to shape and plan for the next phase and learn from the phase that we just worked on” (Researcher04).*

Among the challenges RePORT PAC members faced, one researcher shared the difficulty of engaging in a virtual environment. *“It's kind of interesting because zoom can be very weird, and that sometimes, I'm being honest sometimes things get lost in context or, you know…… you can drift off, look on your phone, you could be looking at another screen and all of those things” (Researcher05).*

### Team dynamics impacting engagement

Three sub-themes were further identified in the qualitative interviews: comfort in contributing, collaborative environment and improvements in online engagement. Comfort in contributing was identified as a theme to describe PAC members' comfort (both PRPs and researchers) in raising concerns and asking questions. This also describes PAC member's perception about feeling heard on the team as well. All PAC members were asked about their comfort in contributing to the team and most PAC members felt comfortable contributing and raising concerns or questions on the team. Some PAC members felt uncomfortable contributing to the project in the beginning. One PRP shared some dissatisfaction with the group dynamic, “…*there's a sense that, oh, you know it doesn't matter what I say because I'm going to get interrupted, or I won't get a chance to speak so in that case I will speak but before doing that I will put my hand up and wait to be called upon” (PRP4).*

In the baseline survey, 4/12 (33%) of respondents strongly agreed that their views were heard on the team while in the follow-up survey, 6/9 (67%) of respondents strongly agreed.

Collaborative environment describes the engagement and involvement of PAC members in the project as well as the influence of the AbSPORU team and principal investigator on the project and team. The theme also encompasses what has been working well in the team and what can be improved on. Most PAC members shared that it was a collaborative environment with the advisory council being very engaged. One researcher shared what they thought could be improved on in terms of collaboration *“I think it just needs to be better communication between all of these working groups and… going back to some sort of a control center that kind of recognizes what each of the working groups are doing as they can communicate with each other.” (Researcher 02).*

All PAC members that participated in the interviews gave suggestions on how to improve engagement in a virtual environment. The main suggestions for improvements were around communication within the team as well as dynamics of the monthly PAC meetings. One PRP suggested *“…it should be almost mandatory to have your camera on and now it's way better if you see people and you interact then it feels more personal…” (PRP5).*

### Supports for PRP to contribute

Respondents of the survey were asked if they had the supports they needed to contribute to the project and 3/12 ( 25%) strongly agreed that the supports were available in the baseline survey while 6/9 (67%) strongly agreed in the follow up survey.

Three subthemes were further identified in the interviews: capacity building opportunities, flexibility and accommodation in involvement and compensation for time and contributions. Most PAC members particularly the PRPs shared appreciation for the training and capacity building opportunities available at various stages of the project. One PRP shared their perspectives of the capacity building opportunities within the team: *“The team was very helpful I have to say so even for encountering… new areas… thematic analysis, for instance, and being involved in putting a code book together and even like going back to the interviews and stuff to be comfortable…the training, the workshops” (PRP5*).

Most PAC members shared that the project was flexible with regards to time commitment and level of contribution. One PRP shared that the project was accommodating to the PRPs *“I think there's been a lot of flexibility for people to like… If you can't make a meeting …at least it's recorded so that you can still watch it” (PRP8).*

Most PRPs were appreciative of the compensation that was given for their contribution and time. One PRP shared how the compensation influenced their life significantly *“…the fact that I had… it was like very small employment, but I had employment during COVID and like some pocket money for ice cream. Basically, actually, all the money went to my dog, she was very expensive… Ah, so the fact I could take her to a vet and get her normal good food, with the money that I get from the study, … I know that not for all the participants, patient partners that compensation is important, but for me, it plays a big role…” (PRP2).*

One researcher shared that the compensation process was challenging initially from the research team’s perspective: *“we have to ensure that everyone is being compensated the way they would like to be compensated; we had to have individual meetings with them. Well, not me, but [Researcher] had to have individual meetings with everybody. So, I think that was initially…the foundations in order were the challenge initially and even now I feel like we are still learning and moving ahead…we see that oh this is not working out, we need to figure something else out and we do feel bad that we're doing a back-and-forth dance.” (Researcher03).*

### Impact of PRP on project

Three sub-themes were identified among PAC members: contributing more than lived experience, input from PRP changes the direction of the project and how it is carried out, and personal impact as well as on the healthcare system. The baseline survey showed that 4/12 (33%) of respondents strongly agreed that the council had a significant impact on the project. The follow-up survey showed that 6/9 (67%) strongly agreed.

More than half of the PAC members, both PRPs and researchers that participated in the interviews felt that the PRPs on the team bring more than lived experience to the project. They bring valuable knowledge, skills as well as personal experiences to the team. One PRP shared an example of their contribution to the team: *“I just remember in our human design process like there was a phrase in one of the transcripts and I recognize that phrase and it's connected to this broader movement and one of the other patients actually thanked me for saying what this thing was all about, cause they haven't heard about it … It's not exclusive to patient knowledge, but it was something that I brought, and I knew about it…” (PRP6).*

Most PAC members, both PRPs and researchers that participated in the interviews felt that the PRPs contributions were impactful to the project. The contributions of PRPs guided the project direction as well as how it was carried out. One researcher shared how PRPs impacted the project recruitment process: *“I think our research changed, just like not big direction, but like changed in terms of how we did things. We still are working towards the same goal, but how we did things, how we interviewed patients, how our script was worded … how we even did the recruitment posters to recruit patients, all were significantly impacted by our patient partners like, perspectives.” (Researcher02).*

Another researcher shared that PRP voices have been valuable in specific areas of the project:* “So, I would say that the places that patient voice has been important. Like I said, is in the data analysis the building of how we collect that data because all of that has been truthfully co-design, they have provided insight in how we collect the data, how we analyze the data, and then how we build whatever comes out of that, whatever the intervention is. So, I'd say those are the three main areas, leveraging their lived experience or their perspectives was good” (Researcher 05).*

Among the reasons PAC members were motivated to be involved in the project, PAC members indicated that the project would have an impact on improving patient experience in the hospital. One PRP expressed their personal motivation for being involved in the project: *“I think what the biggest motivator is for me is people listening. We have a huge problem in our system in healthcare, especially in health care. They do not listen, you go in and you tell the doctors or nurse about something that is wrong, and they just pat on head and said, “OK yeah, hmm” and then they walk away and do something clearly different, well, I think that we have a lot of people listening” (PRP1).*

All PRPs shared that the project has changed them on a personal level. One PRP expressed the personal benefits they experienced as a result of their involvement in the project: *“Yes, […] I definitely can tell my communication skills are better. I understand more about qualitative research. I have more skills in how to make interview guides, how to conduct interviews and how to analyze interviews. I know better how to do human-centred design. I find this very fascinating and interesting” (PRP2).*

### Improving the diversity of the council

More than half of the PAC members that participated in the interviews including PRPs, and researchers shared challenges with the team regarding the diversity of the PAC. Interviews with PAC members revealed varying interpretations of the term 'diversity'. For instance, half of the PAC members shared that the advisory council was not diverse enough. Some PAC members shared ways that the advisory council can be more diverse. One researcher shared, “*People from different backgrounds…would be very helpful […], I think people with different languages also because I know that I have been in part of other research studies and definitely language barrier is definitely one of the things that can be problematic in a hospital” (Researcher03).*

Two PAC members (one researcher and one PRP) shared that it depends on how diversity is defined: *“This was the question because I am not sure by diversity [pause] If it is diversity of health conditions, if it is diversity of socioeconomic professional backgrounds if it is diversity of gender or diversity of age. I am not sure. I think there is a lot of different perspectives and experiences, but I am not sure I know what the diversity word seeks to address or cover” (Researcher06).*

One of the survey questions asked whether respondents agreed or disagreed that the individuals engaging in the RePORT project represent a broad range of perspectives. In the open text question, respondents provided more insight on how the question was interpreted, with some interpreting broad perspectives as health experiences or age group. In the baseline survey, 2/12 (17%) of respondents strongly agreed that there was a broad range of perspectives on the RePORT project while 6/12 (50%) somewhat agreed. However, in the follow-up survey, 3/9 (33%) strongly agreed that there was a diverse range of perspectives on the team. 2/9 (22%) strongly disagreed that there was diversity on the team.

## Discussion

This study provided an in-depth understanding of how PRPs and researchers work together in health research. We found that perceptions and opinions regarding the PAC team’s diversity was mixed. Most PAC members also felt their views and opinions were heard. PAC members expressed that the RePORT PRPs had a valuable impact on the project, and that their contributions helped to inform the project. Furthermore, our findings revealed that the RePORT PRPs appreciated the supports provided, including compensation and training opportunities.

We formally evaluated the patient engagement processes and outcomes using a validated tool, PPEET and semi-structured interviews. Our findings are similar to other studies that have evaluated patient engagement within health research teams. For instance, in a study evaluating patient engagement in health research projects in Newfoundland and Labrador, they found that the involvement of patients influenced the research decisions and participants felt that it improved the research quality and uptake [1]. Previous research has demonstrated limited evidence regarding the quantitative evaluation of patient engagement in health research and its impact on the quality of health research [24]. Our findings contributes valuable insights to the growing body of research on the quantitative evaluation of patient engagement in health research.

Within the RePORT PAC, PRPs actively contributed during monthly PAC meetings. Furthermore, all PAC members were provided with opportunities to collaborate at different stages and aspects of the research cycle, including recruitment, data collection, and analysis. Members of the PAC felt their views, opinions, and concerns were heard during meetings, and a variety of viewpoints were acknowledged. The findings of the baseline survey were shared with the PAC, highlighting areas for improvement within the council. The results of the follow-up survey showed that respondents reported improvements in their views and opinions being heard and the availability of support compared to the baseline survey (Fig. 1). These improvements can be attributed to the development and progression of the project including project duties and meeting agenda. Initially, the PAC team primarily focused on setting up the project, creating engagement documents, and templates. However, as the project progressed, the AbSPORU Patient Engagement Team provided additional support, including research skill-building workshops to the patient partners on the team such as an introduction to semi-structured interviewing and thematic analysis. The team also collaboratively developed a Terms of Reference document through an iterative process, which fostered an environment of mutual respect and equity, promoting positive communication within the team. The findings of this study are consistent with another research study that found most patients and academic researchers both felt they were being heard [25]. Furthermore, another study showed that PRPs were engaged in the study and their contributions were impactful to the research [2]. Overall, most RePORT PAC members found the environment to be collaborative and conducive to open discussion. This type of environment enhanced collaboration and supported PAC members to become more engaged.

The contributions of the RePORT PAC PRPs have been instrumental to the project phases such as the co-development of a recruitment poster, semi-structured interview guide, rapid and thematic data analysis and the co-development of a patient educational tool based on patient participant data. In addition, RePORT PAC PRPs were offered opportunity to collaborate on additional project research activities including data collection, analysis, knowledge translation/mobilization. These findings are in line with another study that showed that the inputs from PRPs influenced the research direction [1]. The study demonstrated that PRPs were impactful in project decisions and desired to take part in data analysis, according to one study [1]. Another study showed that PRPs felt that their contributions to the project influenced the research design and project success [2]. Despite receiving training in thematic data analysis through the delivery of a workshop and supplemental practice activity, some RePORT PAC PRPs were disappointed with not being fully engaged in thematic analysis initially. However, while conducting the evaluation study, other opportunities in data analysis emerged for RePORT PAC PRPs. In addition to influencing the project, priorities and research direction, RePORT PAC PRPs also reported significant personal and professional development as a result of their involvement in the project.

Besides lived experience as a patient, PRPs bring valuable skills, knowledge, and additional research and professional experience that can influence and impact its success. The RePORT PAC PRPs had different professional and personal experiences. There were 3/9 PRPs (44%) who had experience interviewing patients. Two RePORT PAC members and one of the AbSPORU Patient Engagement team members are graduates of the Patient and Community Engagement Research (PACER) training program [26] and two RePORT PAC members were current students.

Compensation for PRPs in health research is a relatively new area and only a few healthcare organizations have guidelines in place for how PRP contributions are valued. The CIHR SPOR Patient Engagement Framework defines support for PRPs as safe environment, training, education, cultural competencies and financial compensation [27]. One study done by patients highlighted five principles for the importance of compensation of PRPs in health research: these included equity, different motivations, respect for vulnerability, commitment, and barrier removal [28]. The study also highlighted the importance of the research team having a conversation with PRPs about the compensation available in the project and suggested that this would also build rapport with PRPs [28]. AbSPORU Patient Partner Appreciation Working Group co-developed a guideline on how to appreciate the time and effort that PRPs provide in health research [29]. Other organizations such as the SPOR Evidence Alliance [30], and Child Bright Network [31] have all set guidelines for patient partner compensation. Apart from compensation, RePORT PAC PRPs were grateful for the several training opportunities available at various stages of the project.

There were mixed responses from PAC members about the range of perspectives on the RePORT project. Diversity and inclusivity are important aspects of health research. PRPs as well as study participants should reflect diversity in areas such as ethnicity, sex, gender, age, as well as various research experience. Our findings are consistent with a recent study examining sociodemographic characteristics of PRPs across multiple healthcare system in Canada; they found that there was lack of diversity with regards to under-represented populations, age, gender, sex etc [32]. The study calls for deeper insights on the recruitment approaches for PRPs and its outcomes [32].While diversity has been acknowledged as important to patient engagement research, to the best of our knowledge, previous papers have not specifically identified it as a theme in interviews conducted with PRPs or research teams. In an evaluation study by Tremblay et al. [33], specific strategies were identified to effectively engage Indigenous patient partners in POR. These strategies encompassed building relationships, capacity building initiatives and accessibility and flexibility in the engagement process. Organizations are moving towards building capacity in research teams to support diversity in patient engagement. Centre for Healthcare innovation developed a framework to support and guide diversity and inclusion engagement initiatives [10]. The BC SPOR SUPPORT Unit’s Tapestry Project developed a set of educational modules to increase diversity and inclusivity in health research [34].

This paper emphasizes the importance of diversity within the research team and particularly among PRPs. Most PAC members in the interview made suggestions for improving diversity on the team. One of the suggestions was to put effort into including more gender, and ethnic diversity on the team. This aligns with the recent work done in collaboration with Albertans4HealthResearch in identifying their priorities to work in health research [35]. In addition to identifying diversity as a priority, the study offered recommendations on engaging PRPs in health research projects [35].

### Strengths and limitations

The RePORT project is a patient-oriented research project with active engagement of PRPs through membership in the PAC as well as through the offering of additional capacity building opportunities such as training and collaboration in participant recruitment strategies, data collection, data analysis and dissemination. One of the ABSPORU Patient Engagement team member is also a PRP and has played an instrumental role in our work and is included as a co-author in the publication of the study. A strength of the paper is the involvement of the ABSPORU Patient Engagement team, whose expertise and support helped guide the study. This study utilized a mixed methods evaluation design to provide insight into the complexity of patient engagement on POR projects. Aside from the PRP perspective, this study also includes researcher perspectives, allowing for a broader and comprehensive understanding of health research partnerships. The evaluation results are fed back to the team for continuous improvement. The team is open to receiving feedback and utilizing it in a collaborative manner to ensure the success of the project.

The patient engagement strategy used in the study is a Canadian strategy based on CIHR framework [27] which can be compared to other frameworks like Patient-Centered Outcomes Research Institute (PCORI) framework [36] in the United States. There are similarities in the fundamental pillars of the CIHR framework to other frameworks which allows for potential generalizability. However, it is essential to acknowledge that certain contextual factors may limit the direct generalizability of our findings beyond the Canadian setting.

One limitation of the study is that our research team may not be similar to other research teams. There may be some differences between the experiences highlighted in the paper and those of other PRPs or research teams. There may also be differences with regard to research aim, context, composition, processes etc. Additionally, not all RePORT PAC members participated in the evaluation, and therefore we may have missed valuable perspectives on barriers and facilitators to engagement. As the surveys were anonymous, we also do not know whether the same respondents answered both surveys and whether the views of individual RePORT PAC members changed over time.

## Conclusion

Our findings show that the research partnership between PRPs and the research team can be of significant value to both the PRPs and the project. We identified what worked well and what didn't, as well as offered recommendations for other research teams working with PRPs. This study adds to the growing research on patient engagement in health research.

## Supplementary Information

**Additional file 1**: Themes identified in the semi structured interviews and their associated quotes. This table contains the themes identified through the semi structured interviews, the description of the themes and some associated quotes.

## Data Availability

All data generated or analyzed during this study are included in this published article [and its supplementary information files].
