# Supplementary material for: Working together in health research: a mixed-methods patient engagement evaluation
Source: Res Involv Engagem. 2023 Aug 1;9:62. doi: 10.1186/s40900-023-00475-w (PMC10394768; doi:10.1186/s40900-023-00475-w)
Supplement: Supplementary file 1 — Additional file 1: Themes identified in the semi structured interviews and their associated quotes. This table contains the themes identified through the semi structured interviews, the description of the themes and some associated quotes. [file 40900_2023_475_MOESM1_ESM.docx]

**Table 4.** Themes identified in the semi structured interviews and their associated quotes.

| **Theme** | **Subtheme** | **Description** | ***Associated Quotes*** |
| --- | --- | --- | --- |
| Communication with team members about project | Role clarity | PAC members’ clarity on their role on the project as well as clarity on expectations and instructions throughout the project | ***“****I did understand that my role was going to be as a member of the team…One of my colleagues is like the chairman and then there's people that generally lead the team , but I did understand that my role would just be a member of the team because I did have other, you know, projects on the side that you know took most of my time, so I did not really get engaged in trying to lead the project or the leadership team of the project. So yeah, I did understand my role as a member”* ***Researcher01***  *“I think I learned a little bit more as I became the chair, the co-chair, what those responsibilities would entail and how I could support with that … It was very, […] very much a learning process in the beginning. I won't, I will I'll be lying if I said our roles were outlined very clearly and distinctly in the beginning because it wasn't. It was very much learning on the job and tweaking as we go so it has and now if you ask me, I can really explain it well but in the beginning, I think there was a little bit of how do we go about this? How do we manage things?”* ***Researcher04***  *“Right, yeah, yeah from the Alberta SPOR support unit patient engagement team, being in a supportive role for both the academic researchers and the patient research partners…”* ***Researcher03***  *“I certainly understood the goal of including patients in the project, like that part I really understood, but again, the specifics of who was doing what, when and how? initially was not sort of at all clear… I think it was a couple of meetings before we sort of started then talking about who was actually going to be doing what, when stuff a little more concretely, and then it became more clear”* ***PRP6*** |
|  | Information sharing | This describes how information was shared about project details as well as among team members in and out of meetings. | ***“****How was it communicated? They emailed the agendas; they email any action items that they need. They verbally shared it in meetings. I think the instructions on what they require and what they hope people to do whether you're an advisor, patient, or research or whatever, I think it's quite clear. They also have a shared drive. What else do they do for communication? If I have an issue, I can reach out to them, to the research team personally and say hey I don't get something, so they're very supportive and their communication. There's multiple ways that they communicate and it's a two-way thing, so they're always available”* ***Researcher05***  *“We kind of created a communication strategy early on. And when we discussed that and we're planning around that we decided it would be best to have one person that was the main point of contact, to lessen confusion to many people, too many emails”* ***Researcher04***  *“it's a very open communication. People are very approachable and so especially the research assistant, the patient engagement lead… were open, always available by email…if you needed more information or you wanted a more personal talk then zoom meetings were set up and time provided and quite often like I said extra time provided [for] training”* ***PRP5*** |
| Team dynamics impacting engagement. | Comfort in contributing | PAC members’ comfort in contributing to the project, raising concerns, and asking questions. This subtheme includes members’ perception about feeling heard on the team. | ***“****I'm very open to discussion... I voice my opinion and I talk when I feel that I have something to offer. I'm quiet when I don't feel that I have something to offer, especially when I saw it going to a territory where I'm not really sure about it but as soon as I figure it out in the in the 15, 20 minutes, I'll start asking questions then, But I want to make sure that I learn and if I don't understand it then I'll ask questions so I feel very comfortable. I'll be honest with you. I feel very comfortable”* ***PRP1***  *“I was comfortable for the parts I did take part in. I never felt like anyone said oh you know your ideas are crazy or anything like that. Like I was actually, I've actually been encouraged to be more, you know, like more involved, but I've struggled with figuring out well how. So uh, but I never felt like I couldn't.”* ***PRP7***  *“I was extremely comfortable. At first though when I started, like when you hear all this patient partners certificate and kind of you know stuff like that you're kind of intimidated and like maybe I shouldn't talk too much because there's people that know too much, like the people that already do this for a living, right? So, there's that intimidation at the beginning”* ***Researcher01*** |
|  | Collaborative environment | Engagement and involvement of PAC members in the project creates a collaborative environment. PAC members also describe what has been working well within the team and what can be improved on. | ***“****I think it's so great to see how first everyone was such strangers and now everyone’s so confident and comfortable with each other. They speak their mind. They really do their work. They're so passionate about this project, that I feel like they go over and beyond what their requirements are****” Researcher03***  ***“****I think they work together well; I think they work hard to try to keep communication. The project has been dynamic and the ways that we would involve the patient partners have sort of been learn as you go and also dynamic, but I think we've done a good job of working together and collaborating and making it the best it can be.”* ***Researcher06***  ***“****In terms of collaboration rest of the team, I think there's great communication that I've been involved in I think at least one sort of meeting prep, like a meeting to prepare for our team meeting… it's very clear to me that everyone is working towards the same end…I'm not finding that you know, different people have their own agenda um, or trying to drive the conversation in a certain direction. I think everyone works really well collaboratively and …voices are heard when needed’* ***PRP4***  *“Almost an orientation that said this is SPOR’s role, this is research role, this is your role or the patient partners role and I think that might have smooth things out a little bit cause there was an element, especially in the first, probably…maybe six weeks when my brain was working overtime as I was trying to figure out what was what and who was who and where I fit that …was the challenge. It did sort itself out, it did come to fruition, and I think it's been very effective in the long run”* ***PRP9*** |
|  | Improvements in online engagement | This describes the ways the team can improve engagement and communication in a virtual environment. | *“You can engage in meetings you know through chat or have a camera on and use audio. So…that's very accommodating, and I find that helpful and it was like critical communication… I think in the meetings, everyone is sort of, you know, chiming in…it works really well I have to say. I don't think I've seen this process be as smooth as I've seen this truly come, yeah”* ***PRP4***  *“So, when people actually meet together for the monthly meetings, I think it's really important to use those other features like breakout rooms where people get to talk and chat or open communication rooms. I think using those features on zoom like that where people have a chance to just talk amongst groups and try to solve a problem which really helped”* ***Researcher02***  *“One way to maybe increase engagement is that they can have the slack channel, because you can even have slack on your mobile phone, so it'll be kind of like a text message for everybody, does anyone having a question or a query or they're doing some work that that could help as well. Kinda, have it open for everybody and if everyone else is having the same problem, they can always problem-solve together, so that's always an option”* ***Researcher03*** |
| Supports for PRPs to contribute. | Capacity building opportunities | Opportunities for PAC members to build capacity in different areas including training for interviewing, opportunities to engage in different phases of the project, data analysis, human centred design. | *“It's hard to beat the RePORT team in terms of the teamwork and what really amazes me there is the opportunities they have given to learn and grow with the team. So far, I haven't encountered even a good research team where you are quite active and involved and being provided so many learning opportuniti****es****”* ***PRP5***  ***“****We held sort of a training session right at the beginning, which was like an overview of patient oriented research and started working together with the Patient Advisory Council to co-develop a kind of reference or kind of like working together guideline and then as we started getting into the project work, we helped to really support this work”* ***Researcher04***  ***“****You don't get the opportunity to have at least some training um and like another project I’m a part of, they said, oh we don't have time for that. So, I think that's what makes this a bit unique in having the ABSPORU team, […], involved is that they offer…some kind of training so that you can do these tasks.”* ***PRP8*** |
|  | Flexibility and accommodation in involvement | PAC members described the RePORT team being flexible and accommodating their needs throughout the project. | *“I like that we have notes and a note-taker… I was never asked to like make meeting notes because they know…would be a struggle for me. Also, I like how when we were doing interviews, nobody pressured me to be more involved, or like do an interview or lead an interview.”* ***PRP2***  *“We have a good understanding of all the different team members and what their roles are, what is expected, but it's especially towards patient partners, it's always given the opportunity to take a break or take it slower if we need…We moved the meeting so often… there's a time difference of an hour between BC and Alberta. But to accommodate patient partners and so I guess the Alberta team had to work half an hour longer”* ***PRP5***  *“When I had to, you know, skip out on a couple meetings because I was in the hospital, no one was you know like we have to kick you out of the program because of this or anything like that. So, I think that just feeling like I have that flexibility to sort of do less if I need to and that there's sort of always more to pick up and work on has been pretty good, so I think time commitment wise I've really appreciated specifically the flexibility.”* ***PRP6*** |
|  | Compensation for time and contributions | This describes how participants were compensated for their time as well as contributions in the project. It also includes challenges associated with compensation. | *“I've been financially compensated at least through December, and I need to submit the rest of that, but that definitely sort of helps as well to just, you know, makes it feel a little bit less like an imposition if it's taking, you know time out of my day or whatever.”* ***PRP6***  *“I think, I think having my time valued is really important to me because I've done numerous hours of other things in patient engagement where I haven't been paid anything and this this project is like no, we value your time, we value everything that you do, and we actually show it through compensating you... We do value your time and you're not just, you know doing this for free”* ***PRP8*** |
| Impact of PRPs on project | Contributing more than lived experience | This describes the skills and knowledge that the PRPs bring to the project. | ***“****there was a phrase in one of the transcripts and I recognize that phrase and it's connected to this more broader movement and one of the other patients actually thanked me for saying what this thing was all about, cause they haven't heard about it and like that's, I mean that, It's not exclusive to patient knowledge, but it was something that I brought and I knew about it and I don't know If, if you weren't informed about it, you wouldn't even have picked up on the phrase you would have just said, oh, they're just saying this, but it was a very specific phrase and very tied to a certain thing.”* ***PRP8***  *“The partners have also been very, very helpful, they bring a wealth of knowledge, you know, because I think they've gone through some program, so they bring a wealth of knowledge. They always make sure to keep us in check in the sense that, you know, when we're vying away from the whole goal of the program, they say, well, this is not acceptable, you can't do this or they try to make sure that things are in line, generally with guidelines and also to that we are on track to achieve the objective, meaning that we want to honestly get patient perspective, you know of their care in the hospital right?”* ***Researcher01***  *“we need to utilize what the patient partners are bringing not utilize, but we need to, we need to appreciate what they can bring to the table and that's their lived experience, that’s their perspective, their opinions on things, so I think that's what we're also learning is how to integrate them in this research process in a way that they really can bring out their lived experience and their perspectives instead of like in rigid research format.”* ***Researcher03***  *“Patient partners still don't just come with the lived experience as a patient. They have life experience, their professional skills and stuff like that and they're bringing this all together and being able to work together like that is very unique, so kudos to the team to approach it this way honestly, and to the university to allow that and also to compensate us for our time.”* ***PRP5*** |
|  | Input from PRP changes the direction of the project and how it is carried out. | This describes the contributions of PRPs guiding the direction of the project as well as how it is executed. | *“I definitely think we have improved the way we [inaudible] communicate with patients. So, advertisement, communication, the interview questions [pause] and how the patient partners are involved in interviewing patients, I think that's very helpful. I think it also helped the PI learn about the value of patient partners and how they can help the research”* ***PRP2***  *“Our patient partners had a significant impact on changing things in a way that I wouldn't have thought about, they really changed their recruitment poster because they pointed out that the wording of the recruitment poster is very clinician centred and not very patient centred and … how we should change that to make it easier or make it more desirable and easier for patient participants to want to participate. Even when we looked at the informed consent protocol and they anticipated difficulties that we didn't. So, in our informed consent we had said we don't anticipate that you will have too many difficulties. But then they [patient partners] said, well, it might generate negative thoughts, it might cause them … recount their negative experience in hospital, and so you should provide them with some support for help and so on. So, all of our informed protocol informed consent protocols and stuff changed with that our questions from our interview script changed quite a bit quite a bit as well in terms of the wording****.****”* ***Researcher02***  *"having the chance to kind of review the interview guide and…like it was a collaborative effort…a group effort like we came up with the interview guide that made more sense, that was more specific to patients and, and I think too having at least we had the chance to code one transcript so that we had multiple people that did that one and that helped form part of the code book that was being developed”* ***PRP8*** |
|  | Personal impact as well as on the healthcare system | This describes the impact the project has had on PRPs personally as well as on the health care system. It includes the PAC members motivation to be involved in the project. | *“I think that that because of what we've done … we are shaping the way patients are going to be more involved and the opportunities that we're going to have as with that involvement, and that does mean that there will be some people that they prefer to edit, and that's what they want to do, perfect! we want them to. We will have people like me that want to do more, that are interested in the methods, in the process and how it happens and are willing to learn and support as much as we can.”* ***PRP9***  ***“****It's been really good; I think I've learned a lot about how to work with patient partners. It has actually been a different experience because I've largely worked with other physician or clinician researchers or health services researchers that it's just a different timeline because they're also available like me on most research days kind of nine to five.”* ***Researcher02***  ***“****Working through the human centered design stuff again from that other perspective has been really cool because that is something that I can bring back to sort of my you know my job and my personal interests and seeing it applied in a very, very different framework from where I've seen it applied previously has been really cool too”* ***PRP6*** |
| Improving the diversity of the council |  | This describes the ways that participants feel the advisory council can be more diverse. It also includes how PAC members define diversity and the challenges within the team related to diversity. | *“I think it's very diverse. I think we have a really, really diverse team, diverse perspectives, diverse backgrounds, diverse professions, in fact, like you know, people that have never done anything in the health care before, there's people that live in the labs, you know, there's physicians, there's clinicians, we have residents you know we have this everybody on the team. So, I think it's very diverse, very, very diverse.”* ***Researcher01***  *“Not as diverse as I always want it to be that we tend to be predominantly white females, With education. We do have two men in our group. I'd like to see more persons of color, more backgrounds, more social and economic. I'd really, I would truly love to see a better balance group in all my [inaudible] groups and we struggle with that all the time. What are we missing? Yeah, there's something we need to fix that because I know we're unbalanced”* ***PRP9***  *“…I think it depends on how diversity is looked at? Is it like sex, gender, race, all of those intersecting or yeah, it's [pause] yeah, some aspects good, some aspects not so good, so depends how it's being measured”* ***PRP8*** |

Abbreviations: PAC, patient advisory council; PRPs, patient research partners
